# Improvement of the inactivated SARS-CoV-2 vaccine potency through formulation in alum/naloxone adjuvant; Robust T cell and anti-RBD IgG responses

**DOI:** 10.22038/IJBMS.2022.63527.14015

**Published:** 2022-05

**Authors:** Melika Haghighi, Akbar Khorasani, Pegah Karimi, Mehdi Mahdavi

**Affiliations:** 1 Advanced Therapy Medicinal Product (ATMP) Department, Breast Cancer Research Center, Motamed Cancer Institute, Academic Center for Education, Culture and Research (ACECR), Tehran, Iran; 2 Recombinant Vaccine Research Center, Tehran University of Medical Sciences, Tehran, Iran; 3 Department of FMD Vaccine Production, Razi Vaccine & Serum Research Institute, Agricultural Research, Education & Extension Organization (AREEO), Karaj, Iran; 4 Immunotherapy Group, The Institute of Pharmaceutical Science (TIPS), Tehran University of Medical Science, Tehran, Iran; # These authors contributed eqully to this work

**Keywords:** Alum Adjuvant, Immune responses, Inactivated SARS-CoV-2 – virus, Naloxone, Vaccine formulation

## Abstract

**Objective(s):**

SARS-CoV-2, emerging as a major threat to public health, has to be controlled through vaccination. Naloxone (NLX), an opioid receptor antagonist, demonstrated its adjuvant activity for microbial vaccines. In this study, inactivated SARS-CoV-2 was developed in the Alum/NLX adjuvant to increase the potency of the inactivated SARS-CoV-2 vaccine.

**Materials and Methods:**

BALB/c mice were immunized on days 0 and 14 with inactivated SARS-CoV-2-Alum, -Alum + NLX 3 mg/kg, -Alum + NLX 10 mg/kg, and -Freund adjuvant, as well as PBS. IFN-γ and IL-4 cytokines and Granzyme-B release were assessed with ELISA. In addition, specific total IgG, IgG1/IgG2a isotypes, and ratio as well as anti-RBD IgG responses were assessed with an optimized ELISA.

**Results:**

SARS-CoV-2-Alum-NLX10 group showed a significant increase in the IFN-γ cytokine response versus SARS-CoV-2-Alum, SARS-CoV-2-Alum-NLX3, and PBS groups. The SARS-CoV-2-Alum-NLX3 group exhibited a significant decrease in IL-4 cytokine versus SARS-CoV-2-Alum. The mice immunized with SARS-CoV-2-Alum-NLX10 showed a significant increase in CTL activity versus SARS-CoV-2-Alum and PBS. In addition, mice immunized with SARS-CoV-2-Alum-NLX3, SARS-CoV-2-Alum-NLX10 and SARS-CoV-2-Freund demonstrated an increase in IgG response, as compared with SARS-CoV-2-Alum and PBS group. Furthermore, all formulations of SARS-CoV-2 vaccines could induce both IgG1 and IgG2a isotypes. But, the IgG2a/IgG1 ratio in SARS-CoV-2-Freund and SARS-CoV-2-Alum-NLX10 revealed an increase as compared with that of the SARS-CoV-2-Alum group. Anti-RBD IgG response in the SARS-CoV-2-Alum-NLX10 group showed a significant increase as compared with the Alum-based vaccine.

**Conclusion:**

Formulation of inactivated SARS-CoV-2 virus in NLX/alum adjuvant improved the potency of humoral and, especially, cellular responses.

## Introduction

SARS-CoV-2, a novel coronavirus, has become a major concern for public health worldwide. The major sources of the disease are currently wild animal hosts and infected patients (1). The genome of the virus was sequenced by researchers in which 86.9% of the genome was the same as the SARS-CoV genome (2). The name was later changed to Corona Virus-2 Severe Acute Respiratory Syndrome (SARS-CoV-2) (3). The virus exhibits less pathogenesis but higher dissemination relative to diseases induced by a previously-identified human coronavirus (4). The global spread of SARS-CoV-2, mainly through respiratory droplets and direct contact, has led to development of several vaccines; however, there are doubts about the potency and safety of some currently-used vaccines (5-8). In order to combat the high transmission risk of a virus, it is crucial to identify the most appropriate targets for vaccine formulation. Spike, appearing to be the most appropriate target**,** is used in several vaccines as an immunogen (6). The vaccination strategy was successful in the prevention of some infectious diseases in communities (9, 10). Despite previous coronavirus epidemics, there is a need for a safe and effective vaccine capable of inducing protective and long-lasting immune responses (11, 12). In vaccine development, selection of an immunogen is critical to combat and eliminate the pathogen in a successful immune response. There is accumulating evidence suggesting that the** s**pike protein, as a surface protein of the SARS-CoV-2 virus, is a suitable choice for vaccine development. Several studies demonstrated that humoral responses against the spike protein with neutralization activity are protective in the experimental infections as well as in the people who recovered from the infection (13, 14). Therefore, induction of humoral immune responses, based on the function of B lymphocytes, is a basis for the development of efficient vaccines. 

Importantly, T cell responses are also important for the induction of other aspects of immune responses. Indeed, T cells can serve as a helper to improve the quality and quantity of humoral immune responses (13, 15, 16). One of the most important components of vaccines is adjuvants that influence the quantity, quality, and pattern of immune responses. In fact, adjuvants are molecules or compounds which have inherent immunomodulatory properties and effectively potentiate host antigen-specific immune responses, when administered in conjunction with an antigen (17, 18).

Naloxone (NLX), an opioid receptor antagonist approved by the FDA, is administered to people with opioid peptide-induced respiratory toxicity (19, 20). A variety of studies demonstrated the adjuvant activity of NLX for microbial vaccines (21, 22). It is demonstrated that NLX, alone or in combination with alum, is able to not only induce strong humoral immune responses but also improve Th1 and IFN-γ cytokine responses (18, 23, 24). NLX seems to improve the immunogenicity and efficacy of vaccines by improving the function of T cells (23, 24). Currently, several vaccines have been approved against COVID-19 infection and are being used in the populations that mainly focused on the neutralization antibodies against the spike protein. Although B lymphocytes are responsible for humoral immune responses, the role of T cells, as a helper for humoral immune responses, is not deniable. 

The present study hypothesized that formulation of inactivated SARS-CoV-2 virus, with a modulating agent influencing T cell functions, may improve the quality and quantity of antibody responses. In this regard, the inactivated SARS-CoV-2 virus was prepared in the alum adjuvant and formulated with two doses of 3 and 10 mg/kg of NLX. After immunization of the experimental mice, different aspects of immune responses were analyzed.

## Materials and Methods

***SARS-CoV-2 virus isolation, propagation, inactivation, and quantification***

A throat swab specimen was prepared from a patient who was positive in real-time PCR (Karaj, Alborz province, Iran) for the SARS-CoV-2 virus. In order to isolate the virus, the sample was transferred to the Vero cell-specific for Coronavirus (CCL-18) in Dulbecco’s Modified Eagle’s Medium (DMEM) supplemented with 10% heat-inactivated fetal bovine serum (FBS). The virus strain was purified by the plaque assay and the first purified clone was passaged three times to obtain an efficient stock. The stock virus showed more than 90% CPE within 48 hr post-infection and a titer of 6.5 to 7 TCID50/ml. 

All experiments in the SARS-CoV-2 isolation, propagation, and inactivation were performed in the biosafety level-III facilities. In order to inactivate the isolated SARS-CoV-2 strain, the virus was inactivated using formalin 0.04% V/V at room temperature for 30 hr. Following clarification, the cell debris was concentrated by ultrafiltration and 8% PEG-6000. The inactivated virus was purified using column chromatography. The purified and inactivated viruses were dialyzed against PBS and passed through 0.22 filters. Subsequently, the protein content was measured using Bradford. The final product of the virus was stored at -70 °C until use (24).

***Vaccine formulations***

The inactivated SARS-CoV-2 virus was used for vaccine formulation in alum hydroxide (Pasteur Institute of Iran) and Freund adjuvants (Sigma, USA; Complete and incomplete Freund adjuvants for the first and second immunizations, respectively). Briefly, 4 µg of the virus in PBS buffer was admixed with 200 µg of alum (for one dose vaccine formulation) and shaken at 100-110 RPM for one hour at room temperature to adsorb on the alum adjuvant. To add NLX to the vaccine formulation, one part of the alum-formulated vaccine was mixed with 200 µg of NLX for each dose (10 mg/kg), as an inactivated SARS-CoV-2 Alum-NLX10 vaccine; for another vaccine formulation, one part of the alum-formulated vaccine was mixed with 60 µg of NLX for each dose (3 mg/kg), as an inactivated SARS-CoV-2 Alum-NLX3 vaccine. 

In addition, inactivated SARS-CoV-2 virus in PBS buffer was mixed with the Freund adjuvant (at v/v of 50/50) and homogenized using a homogenizer to achieve a homogenized suspension. In the end, 200 µl of each vaccine formulation contained 4 µg of the virus.

***Mice***

The male BALB/c mice (six- to 8-week-old, N=50) were provided from Royan Institute of Iran (Tehran, Iran). The mice (20 g body weight at the beginning of the study) were housed for 7 days before the immunization, and allowed access to food and drink *ad libitum* with 12-hr light\dark cycles. All mice handling, immunization, and sampling were in accordance with the Animal Care and Use Protocol of the Razi Vaccine and Serum Research Institute of Iran.

***Experimental groups and immunization***

The mice were randomly assigned to five experimental groups and each one consisted of 10 mice. Mice in groups 1–5 were immunized two times, subcutaneously on days 0 and 14 with 4 µg of inactivated SARS-CoV-2-Alum, inactivated SARS-CoV-2-Alum-NLX3 vaccine, inactivated SARS-CoV-2-Alum-NLX10 vaccine, and inactivated SARS-CoV-2-Freund adjuvant (25, 26), as well as PBS as a control group, respectively. Two weeks after the last immunization, cellular and humoral aspects of immune responses were assessed.

***Spleen cell culture and in vitro stimulation with inactivated SARS-CoV-2 virus***

Fourteen days after the last immunization, the spleens of the experimental mice were aseptically removed and dissected mechanically in sterile cold wash buffer (PBS + FBS 2%). The cell suspension was provided by vigorous pipetting and the samples were centrifuged at 300 g for 5 min, and RBCs were lysed using lysis buffer (0.16M ammonium chloride and 0.17M Tris base). After three-time washing, the cell suspension was adjusted to 3×10^6^ cells/ml in RPMI-1640 (Gibco, Germany) supplemented with 5% FBS, 1mM sodium pyruvate, 4mM L-glutamine, 100 µg/ml streptomycin, and 100IU/ml penicillin. The spleen cell suspension was adjusted to 3×10^6^ cells/ml and one milliliter was seeded into 24-well plates and stimulated with 1 μg/ml of inactivated SARS-CoV-2 virus for 48 hr at 37 °C in 5% CO_2_. Afterward, the culture supernatant was harvested by centrifugation at 5000 RPM/10 min and stored at -70 °C for cytokine measurement.

***ELISA for IFN-γ and IL-4 cytokines***

The supernatant from antigen recalled spleen cells was used for IFN-γ and IL-4 cytokines assay. Commercial ELISA Kits for mouse IFN-γ and IL-4 cytokines (Mabtech, Stockholm, Sweden) were used for the assay. ELISA for IFN-γ and IL-4 cytokines was performed according to the manufacturer’s instructions. The quantity of the cytokines of each individual mouse was presented as pg/ml. In addition, the IFN-γ/IL-4 cytokine ratio of each mouse was calculated by dividing the IFN-γ to IL-4 from each mouse. 

***Cytotoxic T lymphocyte (CTL) activity***

The CTL activity was measured by Granzyme B (Gr-B) release (12, 27). Briefly, 1.5×10^6^ spleen cells in complete medium were cultured in 96-well plates and recalled with 0.2 µg of the inactivated SARS-CoV-2 virus. Some wells were considered without antigen as a negative control for each mouse and the total volume for each well was 200 µl. The plates were then incubated at 37 °C in 5% CO_2 _for 48 hr and then the culture supernatants were harvested for Gr-B assay by commercial ELISA kits according to the company manual (eBioscience, USA). For each individual mouse, the pg/ml of stimulated wells was subtracted from the those of unstimulated wells and considered as net Gr-B release which is a criterion of CTL activity.

***ELISA for specific total IgG and IgG1/IgG2a isotypes***

Specific total IgG antibody responses were determined by an optimized indirect ELISA for SARS-CoV-2, which was developed in our laboratory. Briefly, 100 µl of 0.5 µg of inactivated SARS-CoV-2 in PBS was added into each well of 96-well ELISA Maxisorp plates (Greiner, Germany), and put overnight at 4 °C. The wells were washed with washing buffer (PBS containing 0.1% Tween 20) three times and blocked for 1 hr at 37 °C with blocking buffer (2% skimmed milk in washing buffer). The plates were then washed five times with washing buffer, and 100 µl of 1/25 of diluted serum samples (up to 16 serial dilutions) was added into each well and incubated at 37 °C for 2 hr. The wells were washed five times with washing buffer and incubated for 90 min with 100 µl of 1/8000 dilution of Rabbit anti-mouse IgG conjugated to HRP (Razirad, Iran). The wells were washed five times and incubated with 100 µl of TMB substrate in the dark for 30 min. Subsequently, the reaction was stopped by adding 2N H_2_SO_4_ and the color density was read at A_450_ nm with an ELISA reader. Furthermore, specific IgG1 and IgG2a isotypes were assessed using goat anti-mouse IgG1and IgG2a secondary antibodies (Sigma, USA) on 1/25 serum dilutions according to the manufacturer’s manual. 

***ELISA for specific IgG against RBD protein***

The potency of specific IgG antibodies developed against inactivated SARS-CoV-2 vaccine was assessed against RBD protein using an optimized indirect ELISA (28). In order to coat antigens, 100 µl of 2 µg/ml of recombinant RBD protein (The Native Antigen Company, UK) in carbonate-bicarbonate buffer (pH 9.6) was dispensed into 96-well ELISA Maxisorp plates (Greiner, Germany). The plates were incubated overnight at 4 °C and then washed with washing buffer 5 times and subsequently blocked by blocking buffer for 60 min at 37 °C (1.5% BSA in PBS + 0.05% tween 20). The plates were washed with washing buffer and 100 µl of 1/50 serum dilution of all the experimental mice were added to the plates and incubated at 37 °C for 90 min. The plates were washed and 100 µl of 1/8000 dilution of anti-mouse IgG HRP-conjugate (Razirad, Iran) was added to the wells for 90 min. The wells were washed 6 times and incubated with 100 µl of the TMB substrate in the dark for 10 min and the reaction was stopped using 100 µl of 2N HCL. The color density of the plates was measured at A_450_ nm with an ELISA reader. The row data of serum samples of the sham group was used to calculate the cutoff of RBD-ELISA by the equation: Mean + 3SD. The specific IgG response to RBD was presented as OD of RBD ELISA of individual mouse/cutoff.

***Statistical analysis***

The data of immunoassay was presented as mean ± standard deviation (SD). The statistical analysis among the experimental groups was performed using ordinary one-way ANOVA followed by the Tukey test (Graph Pad Prism 6.01 software, La Jolla, CA, USA). In addition, statistical analysis of IgG1, IgG2a isotypes antibodies, as well as the IgG2a/IgG1 ratio, was performed by the Mann-Whitney U test. Among the experimental groups, *P*-values less than 0.05 were considered a significant difference. 

## Results

***IFN-γ cytokine response***

**Figure 1:**
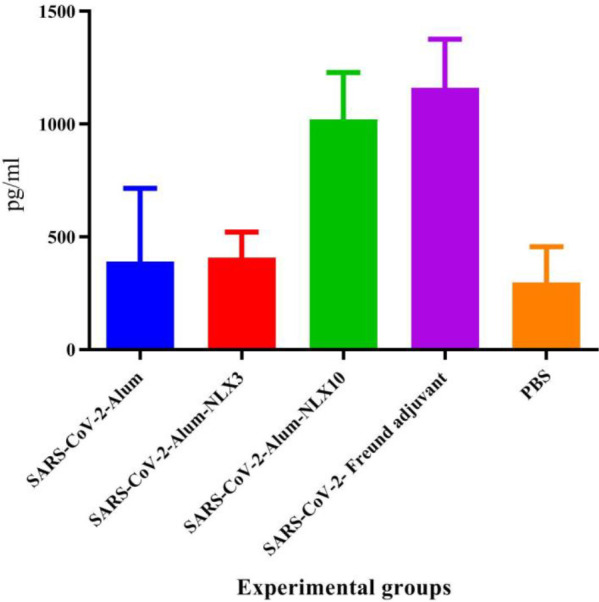
IFN-γ response in the vaccinated mice. Mice immunized with SARS-CoV-2-Alum-NLX10 showed a significant increase in the IFN-γ cytokine secretion versus those immunized with SARS-CoV-2-Alum (*P*=0.0001)

Inactivated SARS-CoV-2-Freund group, as well as SARS-CoV-2-Alum-NLX-10, showed a significant increase in the IFN-γ cytokine response versus the control group (*P*=0.0001). However, mice immunized with the SARS-CoV-2-Alum vaccine and SARS-CoV-2-Alum-NLX3 did not show a significant difference versus the control group (*P*>0.8384). In addition, mice immunized with SARS-CoV-2-Alum-NLX10 showed a significant increase in the IFN-γ cytokine secretion as compared with the SARS-CoV-2-Alum group (*P*=0.0001), while SARS-CoV-2-Alum-NLX3 showed a tiny increase**,** as compared with the SARS-CoV-2-Alum group (*P*=0.9998). Immunization with SARS-CoV-2-Freund showed a significant increase as compared with SARS-CoV-2-Alum and SARS-CoV-2-Alum-NLX3 groups (*P*=0.0001); however, a comparable IFN-γ response was observed in SARS-CoV-2-Alum-NLX10 and SARS-CoV-2- Freund groups (*P*=0.6195) (Figure 1).

***IL-4 cytokine response***

**Figure 2:**
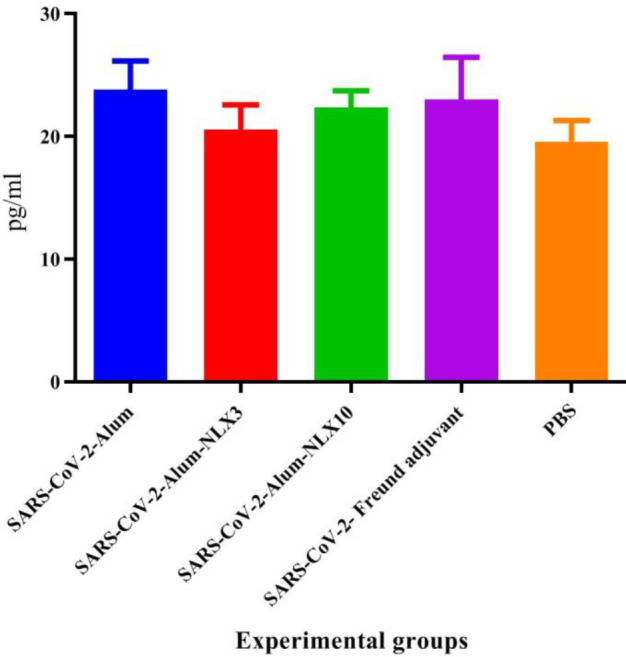
IL-4 response in the vaccinated groups. Naloxone formulated in the vaccine resulted in a significant decrease in the IL-4 response in the SARS-CoV-2-Alum-NLX3 group versus SARS-CoV-2-Alum (*P*=0.0133), while SARS-CoV-2-Alum-NLX10 group showed a slight decrease in the IL-4 response versus SARS-CoV-2-Alum (*P*=0.5631)

Mice immunized with inactivated SARS-CoV-2-Freund as well as SARS-CoV-2-Alum showed a significant increase in the IL-4 cytokine response versus the control group (*P*=0.0109 and *P*=0.0010, respectively). Immunization with SARS-CoV-2-Alum-NLX3 showed a significant increase versus the SARS-CoV-2-Alum group (*P*=0.0133), while the SARS-CoV-2-Alum-NLX10 group showed a slight decrease versus the SARS-CoV-2-Alum group (*P*=0.5631) (Figure 2).

***IFN-γ/IL-4 ratio***

**Figure 3.**
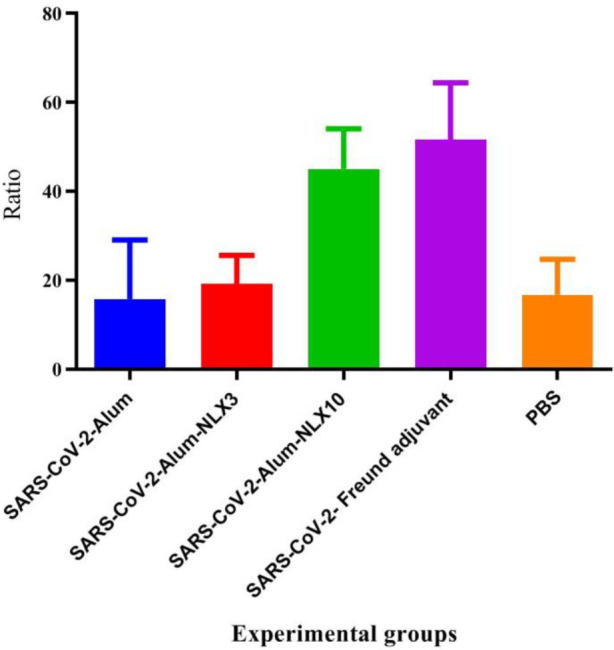
IFN-γ/IL-4 ratio after vaccination of the study groups. Mice immunized with SARS-CoV-2-Alum-NLX10 showed a significant increase in the IFN-γ/IL-4 ratio, as compared with those immunized with SARS-CoV-2-Alum and SARS-CoV-2-Alum-NLX3, as well as PBS groups (*P*<0.0001). However, the SARS-CoV-2-Alum-NLX3 group revealed a 21.46% increase in the IFN-γ/IL-4 ratio versus the SARS-CoV-2-Alum group (*P*=0.9519)

Results from the IFN-γ/IL-4 ratio (Figure 3) demonstrated that immunization with SARS-CoV-2-Freund resulted in a significant increase versus SARS-CoV-2-Alum, SARS-CoV-2-Alum-NLX3**,** and PBS groups (*P*<0.0001). Furthermore, mice immunized with SARS-CoV-2-Alum-NLX10 revealed a significant increase, as compared with SARS-CoV-2-Alum**,** SARS-CoV-2-Alum-NLX3**,** and PBS groups (*P*<0.0001), while the SARS-CoV-2-Alum-NLX3 group showed a 21.46% increase as compared with the SARS-CoV-2-Alum group (*P*=0.9519). 

***Granzyme-B release***

**Figure 4:**
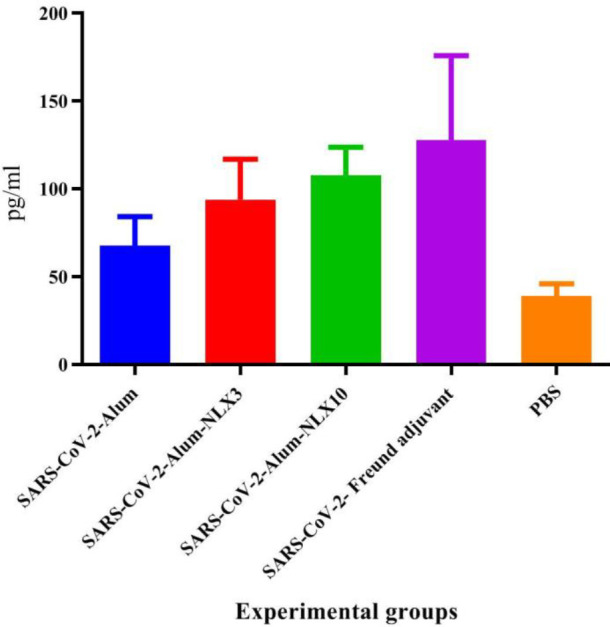
Granzyme-B release of vaccinated mice as a criterion of CTL activity. Mice injected with SARS-CoV-2-Alum-NLX10 and SARS-CoV-2-Freund showed a significant increase in Gr-B release versus the SARS-CoV-2-Alum group (*P*=0.0216 and *P*=0.0002, respectively)

Results from CTL activity based on Granzyme-B release (Figure 4) showed that mice injected with SARS-CoV-2-Freund, SARS-CoV-2-Alum-NLX10**,** and SARS-CoV-2-Alum-NLX3 adjuvant had a significant increase**,** as compared with the control group (*P*<0.0011), while SARS-CoV-2-Alum group did not show a significant difference versus the control group (*P*=0.1871). Furthermore, the mice immunized with SARS-CoV-2-Alum-NLX10 and SARS-CoV-2-Freund demonstrated a significant increase in Gr-B release versus the SARS-CoV-2-Alum group (*P*=0.0216 and *P*=0.0002, respectively). Mice immunized with SARS-CoV-2-Freund and SARS-CoV-2-Alum-NLX10 indicated increased Gr-B release, as compared with SARS-CoV-2-Alum-NLX3 groups (*P*=0.0706 and *P*=0.7986, respectively).

***Specific total IgG response***

**Figure 5:**
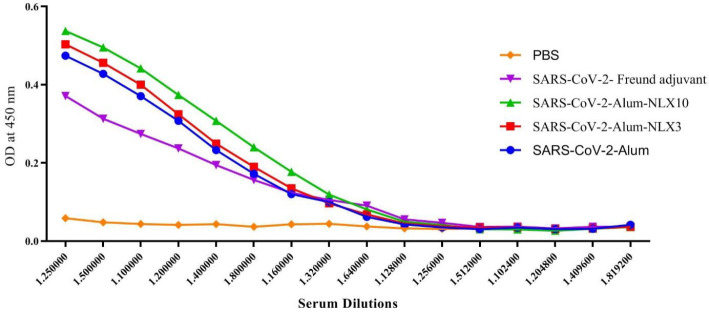
Specific IgG response in the vaccinated mice after two times immunization. Injection with SARS-CoV-2-Alum and also SARS-CoV-2-Alum-NLX3 vaccine revealed a significant increase in specific IgG versus SARS-CoV-2-Freund at dilutions of 1/25 up to 1/200 (*P*<0.0129). In addition, injection with SARS-CoV-2-Alum-NLX10 revealed a significant increase versus the SARS-CoV-2-Freund group at dilutions of 1/25 up to 1/800 (*P*<0.0011). Naloxone formulated in the vaccine resulted in a significant IgG response in the SARS-CoV-2-Alum-NLX10 group versus the SARS-CoV-2-Alum group at dilutions of 1/25 up to 1/800 (*P*<0.0354). Furthermore, the SARS-CoV-2-Alum-NLX3 group revealed a borderline increase versus SARS-CoV-2-Alum (*P*>0.0527)

As shown in Figure 5, the mice immunized with SARS-CoV-2-Alum-NLX3 and SARS-CoV-2-Alum showed a significant increase in IgG response versus the control group at dilutions of 1/25 up to 1/1600 (*P*<0.0063), while SARS-CoV-2-Alum-NLX10 and SARS-CoV-2-Freund groups exhibited a significant IgG response at dilutions of 1/25 up to 1/3200**,** as compared with the control group (*P*<0.0463). Mice immunized with SARS-CoV-2-Alum and SARS-CoV-2-Alum-NLX3 showed a significant increase**,** as compared with the SARS-CoV-2-Freund group at dilutions of 1/25 up to 1/200 (*P*<0.0129). In addition, SARS-CoV-2-Alum-NLX10 showed a significant IgG response**,** as compared with the SARS-CoV-2-Freund group at dilutions of 1/25 up to 1/800 (*P*<0.0011). Furthermore, the SARS-CoV-2-Alum-NLX10 group revealed a significant increase**,** as compared with SARS-CoV-2-Alum at dilutions of 1/25 up to 1/800 (*P*<0.0354), while SARS-CoV-2-Alum-NLX3 group exhibited no significant differences versus SARS-CoV-2-Alum and SARS-CoV-2-Alum-NLX10 groups in any dilutions (*P*>0.0527).

***Specific IgG1 isotype***

**Figure 6:**
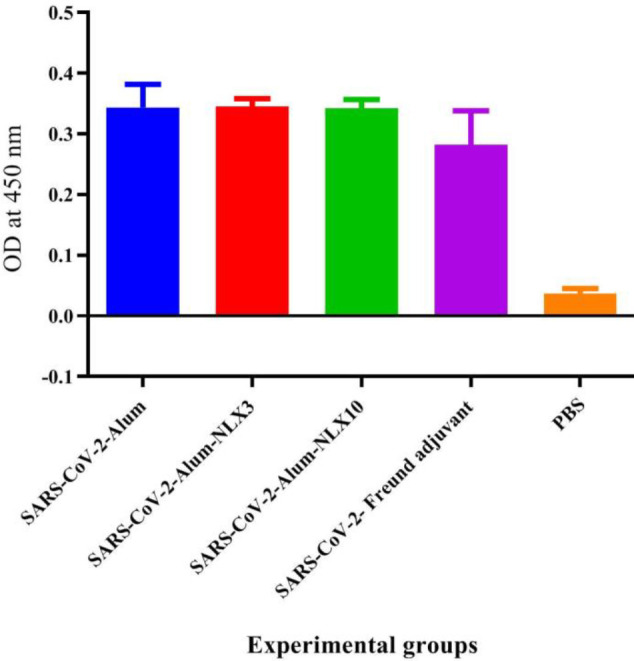
Specific IgG1 isotype antibodies after two shots of vaccine. Mice immunized with SARS-CoV-2-Freund, SARS-CoV-2-Alum, SARS-CoV-2-Alum-NLX3, and SARS-CoV-2-Alum-NLX10 demonstrated a significant difference versus the control group (*P*<0.0001). Mice immunized with SARS-CoV-2-Alum, SARS-CoV-2-Alum-NLX3 and SARS-CoV-2-Alum-NLX10 exhibited a significant difference versus the SARS-CoV-2-Freund group (*P*<0.0002)

Results from specific IgG1 isotype antibodies (Figure 6) demonstrated that SARS-CoV-2-Freund, SARS-CoV-2-Alum, SARS-CoV-2-Alum-NLX3**,** and SARS-CoV-2-Alum-NLX10 revealed a significant increase**,** as compared with the control group (*P*<0.0001). Mice immunized with SARS-CoV-2-Alum, SARS-CoV-2-Alum-NLX3**,** and SARS-CoV-2-Alum-NLX10 revealed a significant increase**,** as compared with the SARS-CoV-2-Freund group (*P*<0.0002). Mice immunized with SARS-CoV-2-Alum-NLX10 and SARS-CoV-2-Alum-NLX3 did not show significant differences versus SARS-CoV-2-Alum (*P*>0.4181).

***Specific IgG2a isotype***

**Figure 7:**
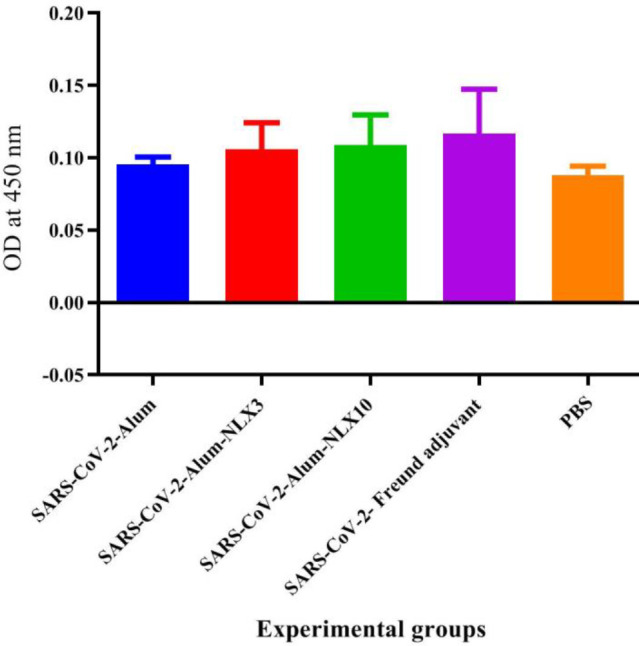
IgG2a isotype antibodies in mice after two times vaccination. Mice immunized with SARS-CoV-2-Freund, SARS-CoV-2-Alum, SARS-CoV-2-Alum-NLX3, and SARS-CoV-2-Alum-NLX10 showed an increase in the IgG2a response versus the control group (*P*=0.0157, *P*=0.0664, *P*=0.0309 and *P*=0.0064, respectively). Mice immunized with SARS-CoV-2-Alum-NLX3 and SARS-CoV-2-Alum-NLX10 had increased IgG2a isotype versus SARS-CoV-2-Alum but statistically it was not significant (P=0.7354 and P=0.1941, respectively)

Results from specific IgG2a isotype antibodies in SARS-CoV-2-Freund, SARS-CoV-2-Alum, SARS-CoV-2-Alum-NLX3, and SARS-CoV-2-Alum-NLX10 showed an increase in the IgG2a versus the control group (*P*=0.0157, *P*=0.0664, *P*=0.0309, and *P*=0.0064, respectively). Mice immunized with SARS-CoV-2-Alum-NLX3 and SARS-CoV-2-Alum-NLX10 increased the IgG2a versus the SARS-CoV-2-Alum group; however, this was not statistically significant (*P*=0.7354 and *P*=0.1941, respectively) (Figure 7).

***IgG2a/IgG1ratio***

**Figure 8:**
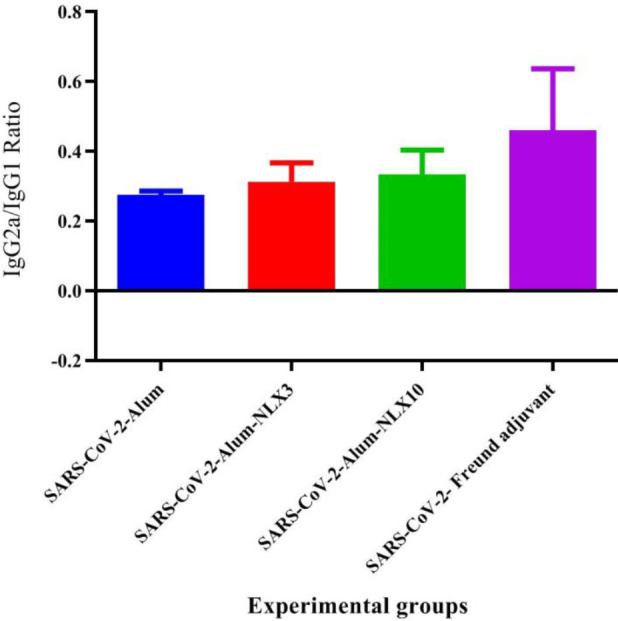
IgG2a/IgG1 ratio of experimental mice groups. Mice injected with SARS-CoV-2-Freund and SARS-CoV-2-Alum-NLX10 showed an increase versus the SARS-CoV-2-Alum group (*P*=0.0006 and *P*=0.0262, respectively)

The results from the IgG2a/IgG1 ratio in SARS-CoV-2-Freund, SARS-CoV-2-Alum-NLX3 and SARS-CoV-2-Alum-NLX10 exhibited an increase in the IgG2a/IgG1ratio versus the SARS-CoV-2-Alum group (67.13%, 13.45% and 20.96%, respectively; *P*=0.0006, *P*=0.4767, and *P*=0.0262, respectively)(Figure 8).

***Specific IgG against the RBD protein***

**Figure 9:**
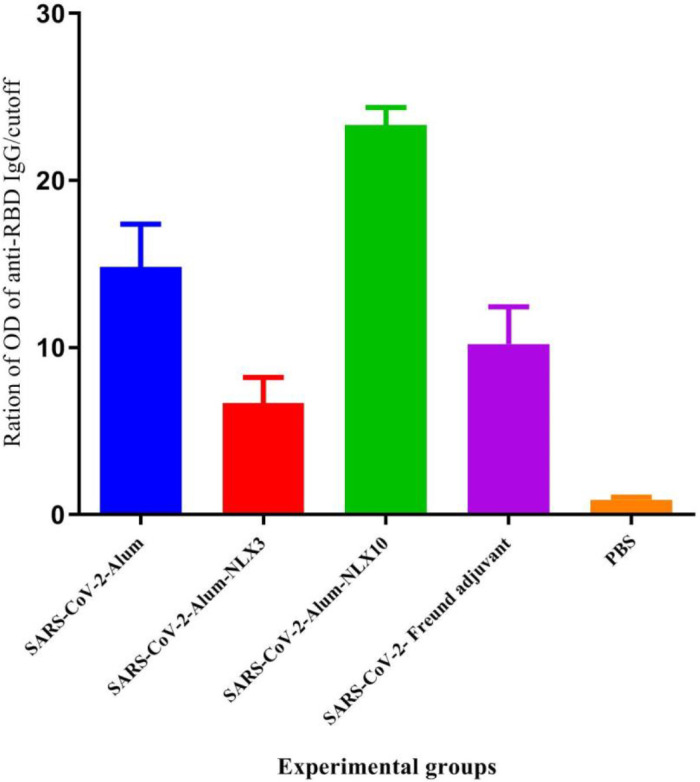
Specific IgG antibody response against RBD protein. The IgG response to RBD presented as OD of RBD ELISA /cutoff for each individual mouse. Results demonstrated that mice injected with SARS-CoV-2-Alum-NLX3 exhibited a significant decrease in anti-RBD IgG response versus the SARS-CoV-2-Alum group (*P*=0.0001). However, the SARS-CoV-2-Alum-NLX10 group revealed a significant increase, as compared with those immunized with SARS-CoV-2-Alum and even SARS-CoV-2-Freund (*P*<0.0001)

The results in SARS-CoV-2-Freund, SARS-CoV-2-Alum, SARS-CoV-2-Alum-NLX3**,** and SARS-CoV-2-Alum-NLX10 groups showed a significant increase against the RBD protein, as compared with the control group (*P*<0.0001). In addition, the SARS-CoV-2-Alum-NLX3 group showed a significant decrease in anti-RBD IgG response, versus the SARS-CoV-2-Alum group (*P*=0.0001**). **Nevertheless, the SARS-CoV-2-Alum-NLX10 group revealed a significant increase**,** as compared with those immunized with SARS-CoV-2-Alum and even SARS-CoV-2-Freund (*P*<0.0001) (Figure 9).

## Discussion

The unexpected appearance of SARS-CoV-2 and its rapid spread endanger the public health and the economies of all countries worldwide. Although tremendous attempts were made to curb the virus’s spread, most of them were not successful in the control of the infection (29, 30). In this light, the vaccines developed by DNA and mRNA-based technologies, viral vector, inactivated organism, live-attenuated, and recombinant vaccines have been used for prevention of the infection (8, 30-32). Eventually, several vaccines were approved and commercialized for human use and are now being used in various countries. These vaccines mainly target the spike proteins and are designated based on humoral immune responses as well as, somehow, T cell responses. Inactivated virus vaccine is one of the approved vaccines for the SARS-CoV-2 virus, which showed lower potency as compared with mRNA and subunit vaccines (33). Therefore, there is an urgent need for improvement of immune responses in this type of vaccine.

In the present study, we hypothesized that changes in the vaccine formulation toward a robust T cell response may influence the vaccine potency. In this regard, NLX, as an immunomodulator, was used in this study because of the fact that NLX could modulate T cell responses in the various vaccine models (18, 21, 23, 24).

Results from IFN-γ cytokine responses in the SARS-CoV-2-Alum-NLX10 group showed a significant increase as compared with alum-based and, even, SARS-CoV-2-Alum-NLX3 vaccines. This finding showed that polarization of T cell responses toward the Th1 pattern is triggered when the vaccine is formulated with NLX. In addition, polarization toward the Th1 pattern seemed to be dose-dependent because the dose of 10 mg/kg was more potent than that of 3 mg/kg. Our previous studies on the adjuvant activity of NLX in several vaccine models confirmed this finding. Th1 polarization was first confirmed in the HSV DNA vaccine (18) and then in HPV (24), HIV-1 (23, 26), and several bacterial vaccine models (22, 34, 35). It is well-known that the Th1 response is highly critical in the control and clearance of viral infections (12), which is a characteristic of NLX in the modulation of immune responses. The results from IL-4 cytokine secretion in the SARS-CoV-2-Alum-NLX3 group showed a significant decrease, but not in SARS-CoV-2-Alum-NLX10. Suppression of the IL-4 response in the SARS-CoV-2-Alum-NLX3 group is another evidence for modulation toward the Th1 pattern when compared with the Alum-based vaccines. Consistent with our findings, previous studies revealed that NLX has the ability to suppress the IL-4 cytokine response (18, 23, 36), which is another confirmation of Th1 polarization. Furthermore, the IFN-γ/IL-4 ratio in mice immunized with SARS-CoV-2-Alum-NLX10 showed a significant increase, as compared with SARS-CoV-2-Alum, confirming strong Th1 polarization through the vaccine formulated in NLX. The cytokine ratio in mice immunized with SARS-CoV-2-Alum-NLX10 is comparable with SARS-CoV-2-Freund, showing the ability of NLX in stimulation of T cells and shifting toward the Th1 pattern as reported by previous studies (18, 34). 

The activity of TCD8+, based on Gr-B release, showed that NLX could improve the CTL activity in the SARS-CoV-2-Alum-NLX10 group, as compared with the alum-based vaccine. In addition, NLX10 seemed to be more potent than NLX3 in the vaccine formulation for the induction of CTL activity. This finding showed the potency of NLX in the improvement of CTL activity. In addition, this effect was demonstrated to be dose-dependent similar to those detected in the polarization toward the Th1 pattern. A study conducted on a murine cancer vaccine model also showed that NLX, in combination with this vaccine, improved the CTL response, which is consistent with our study (37).

Assessment of the specific IgG antibody titer in the SARS-CoV-2-Alum-NLX10 group exhibited a significant increase versus the SARS-CoV-2-Alum group, while NLX3 showed no dramatic effect. This finding showed the potency of NLX in improvement of humoral immune responses; importantly, this effect was dose-dependent because NLX10, in contrast to NLX3, was a more successful dose in the improvement of the IgG response versus the vaccine. In addition, results from IgG1 and IgG2a isotypes showed the potency of NLX in improvement of the IgG2a isotype and IgG2a/IgG1 ratio, which is a criterion of the Th1 pattern because the IFN-γ cytokine is the causative of isotype switching of IgM to IgG2a class (28, 38). Of note, this result is parallel to the IFN-γ cytokine response, confirming the improvement of the Th1 immune response.

Several studies demonstrated that NLX reinforced humoral immune responses in the vaccine formulation, as our findings achieved in the inactivated SARS-CoV-2 vaccine model (22, 23, 26, 39). It is well-known that antibodies in vaccinations and infections are produced by B cells but it is well-known that the function, as well as the quantity and quality of antibody responses, highly depend on the help of T cells through cytokines, receptor-ligand contacts, and growth factors (40, 41). Herein, NLX seemed to provide a helper signal for B cell responses in the vaccine formulation through improving T cell responses and thereby improved humoral immune responses (42). Antibody response is the first barrier in the SARS-CoV-2 virus neutralization and disease prevention. NLX, as an adjuvant in the vaccine formulation, resulted in a dramatic humoral response, highlighting the potency of this adjuvant in the inactivated SARS-CoV-2 vaccine formulation and encouraging human vaccine development.

Next, the anti-RBD IgG response was evaluated, which can potentially demonstrate the neutralization activity (43, 44). Our results showed that NLX10 in the vaccine formulation significantly increased the anti-RBD IgG response while NLX3 suppressed the response in comparison to the alum-based vaccine. This finding showed another potency of NLX in the induction of the antibody response against the neutralizing protein on the virus but the dose of NLX is a critical factor in the adjuvant activity.

## Conclusion

Results from the present study provided evidence for the potency of the NLX/alum adjuvant in the inactivated vaccine model for SARS-CoV-2 which increased T cell and antibodies responses in a dose-dependent manner for NLX. Because of the lower potency of the inactivated vaccine for SARS-CoV-2 in the induction of cellular immune response, this formulation can be used to solve this problem (45, 46). Findings from the present study showed that NLX**,** in combination with Alum, can result in an appropriate inactivated SARS-CoV-2 vaccine, which triggered more robust immune responses in comparison to the alum-formulated vaccine.

## Authors’ Contributions

MM Conceived the study and design; MH, PK, and MM Performed animal handling, assay, data analysis, and manuscript preparation; MM and AK Revised the paper; MEM and AK Supervised the project; MH, PK, AK, and MM Approved the final version of the manuscript.

## Conflicts of Interest

The authors declare that no conflicts of interest exist for this research.

## References

[1] Shi Y, Wang G, Cai XP, Deng JW, Zheng L, Zhu HH (2020). An overview of COVID-19. J Zhejiang Univ Sci B.

[2] Chang L, Yan Y, Wang L (2020). Coronavirus disease 2019: Coronaviruses and blood safety. Transfus Med Rev.

[3] The Lancet Infectious D (2020). Challenges of coronavirus disease 2019. Lancet Infect Dis.

[4] Dhama K, Khan S, Tiwari R, Sircar S, Bhat S, Malik YS (2020). Coronavirus disease 2019-COVID-19. Clin Microbiol Rev.

[5] Alkandari D, Herbert JA, Alkhalaf MA, Yates C, Panagiotou S (2021). SARS-CoV-2 vaccines: Fast track versus efficacy. Lancet Microbe.

[6] Iqbal Yatoo M, Hamid Z, Parray OR, Wani AH, Ul Haq A, Saxena A (2020). COVID-19-recent advancements in identifying novel vaccine candidates and current status of upcoming SARS-CoV-2 vaccines. Hum Vaccin Immunother.

[7] Dhama K, Sharun K, Tiwari R, Dadar M, Malik YS, Singh KP (2020). COVID-19, an emerging coronavirus infection: Advances and prospects in designing and developing vaccines, immunotherapeutics, and therapeutics. Human vaccines & immunotherapeutics..

[8] Jafari A, Danesh Pouya F, Niknam Z, Abdollahpour-Alitappeh M, Rezaei-Tavirani M, Rasmi Y (2022). Current advances and challenges in COVID-19 vaccine development: from conventional vaccines to next-generation vaccine platforms. Mol Biol Rep.

[9] Najminejad H, Kalantar SM, Mokarram AR, Dabaghian M, Abdollahpour-Alitappeh M, Ebrahimi SM (2019). Bordetella pertussis antigens encapsulated into N-trimethyl chitosan nanoparticulate systems as a novel intranasal pertussis vaccine. Artif Cells Nanomed Biotechnol.

[10] Amini Y, Tebianian M, Mosavari N, Fasihi Ramandi M, Ebrahimi SM, Najminejad H (2017). Development of an effective delivery system for intranasal immunization against Mycobacterium tuberculosis ESAT-6 antigen. Artif Cells Nanomed Biotechnol.

[11] Corey L, Mascola JR, Fauci AS, Collins FS (2020). A strategic approach to COVID-19 vaccine R&D. Science.

[12] Mahdavi M, Ebtekar M, Khorshid HRK, Azadmanesh K, Hartoonian C, Hassan ZM (2011). ELISPOT analysis of a new CTL based DNA vaccine for HIV-1 using GM-CSF in DNA prime/peptide boost strategy: GM-CSF induced long-lived memory responses. Immunology letters.

[13] Wang J, Peng Y, Xu H, Cui Z, Williams RO (2020). The COVID-19 vaccine race: challenges and opportunities in vaccine formulation. AAPS PharmSciTech.

[14] Kuo T-Y, Lin M-Y, Coffman RL, Campbell JD, Traquina P, Lin Y-J (2020). Development of CpG-adjuvanted stable prefusion SARS-CoV-2 spike antigen as a subunit vaccine against COVID-19. Scientific Reports.

[15] García-Arriaza J, Garaigorta U, Pérez P, Lázaro-Frías A, Zamora C, Gastaminza P (2021). COVID-19 vaccine candidates based on modified vaccinia virus ankara expressing the SARS-CoV-2 spike protein induce robust t-and b-cell immune responses and full efficacy in mice. J Virol.

[16] Cox RJ, Brokstad KA (2020). Not just antibodies: B cells and T cells mediate immunity to COVID-19. Nat Rev Immunol.

[17] Beyer WEP, Palache AM, Reperant LA, Boulfich M, Osterhaus A (2020). Association between vaccine adjuvant effect and pre-seasonal immunity Systematic review and meta-analysis of randomised immunogenicity trials comparing squalene-adjuvanted and aqueous inactivated influenza vaccines. Vaccine..

[18] Jamali A, Mahdavi M, Hassan ZM, Sabahi F, Farsani MJ, Bamdad T (2009). A novel adjuvant, the general opioid antagonist naloxone, elicits a robust cellular immune response for a DNA vaccine. Int Immunol.

[19] Burris S, Norland J, Edlin BR (2001). Legal aspects of providing naloxone to heroin users in the United States. International Journal of Drug Policy.

[20] Yasaghi M, Mahdavi M (2016). Potentiation of human papilloma vaccine candidate using naloxone/alum mixture as an adjuvant: increasing immunogenicity of HPV-16E7d vaccine. Iran J Basic Med Sci.

[21] Jamali A, Mahdavi M, Shahabi S, Hassan ZM, Sabahi F, Javan M (2007). Naloxone, an opioid receptor antagonist, enhances induction of protective immunity against HSV-1 infection in BALB/c mice. Microb Pathog.

[22] Jazani NH, Karimzad M, Mazloomi E, Sohrabpour M, Hassan ZM, Ghasemnejad H (2010). Evaluation of the adjuvant activity of naloxone, an opioid receptor antagonist, in combination with heat-killed Listeria monocytogenes vaccine. Microbes Infect.

[23] Velashjerdi Farahani S, Reza Aghasadeghi M, Memarnejadian A, Faezi S, Shahosseini Z, Mahdavi M (2016). Naloxone/alum mixture a potent adjuvant for HIV-1 vaccine: induction of cellular and poly-isotypic humoral immune responses. Pathog Glob Health.

[24] Kaffashi A, Huang J, Bairami A, Fallah Mehrabadi MH, Yaslianifard S, Bashashati M (2021). Complete genome sequencing and molecular characterization of SARS-COV-2 from COVID-19 cases in Alborz province in Iran. Heliyon.

[25] Fathi M, Nezamzadeh R, Abdollahpour‐Alitappeh M, Yazdi MH, Khoramabadi N, Mahdavi M (2021). Formulation of a recombinant HIV‐1 polytope candidate vaccine with naloxone/alum mixture: Induction of multi‐cytokine responses with a higher regulatory mechanism. APMIS.

[26] Mojarab S, Shahbazzadeh D, Moghbeli M, Eshraghi Y, Bagheri KP, Rahimi R (2020). Immune responses to HIV-1 polytope vaccine candidate formulated in aqueous and alcoholic extracts of Propolis: Comparable immune responses to Alum and Freund adjuvants. Microbial Pathogenesis.

[27] Mahdavi M, Tajik AH, Ebtekar M, Rahimi R, Adibzadeh MM, Moozarmpour HR (2017). Granulocyte‐macrophage colony‐stimulating factor, a potent adjuvant for polarization to Th‐17 pattern: an experience on HIV‐1 vaccine model. Apmis..

[28] Pi-Estopiñan F, Pérez MT, Fraga A, Bergado G, Díaz GD, Orosa I (2022). A cell-based ELISA as surrogate of virus neutralization assay for RBD SARS-CoV-2 specific antibodies. Vaccine.

[29] Hellewell J, Abbott S, Gimma A, Bosse NI, Jarvis CI, Russell TW (2020). Feasibility of controlling COVID-19 outbreaks by isolation of cases and contacts. Lancet Global Health.

[30] Forni G, Mantovani A (2021). Covid-19 vaccines: Where we stand and challenges ahead. Cell Death Differ.

[31] Liu X, Liu C, Liu G, Luo W, Xia N (2020). Covid-19: Progress in diagnostics, therapy and vaccination. Theranostics.

[32] Lazarus JV, Ratzan SC, Palayew A, Gostin LO, Larson HJ, Rabin K (2021). A global survey of potential acceptance of a Covid-19 vaccine. Nat Med.

[33] Kim JH, Marks F, Clemens JD (2021). Looking beyond Covid-19 vaccine phase 3 trials. Nat Med.

[34] Jazani NH, Parsania S, Sohrabpour M, Mazloomi E, Karimzad M, Shahabi S (2011). Naloxone and alum synergistically augment adjuvant activities of each other in a mouse vaccine model of Salmonella typhimurium infection. Immunobiology.

[35] Khorshidvand Z, Shahabi S, Mohamadzade H, Daryani A, Tappeh KH (2016). Mixture of alum–naloxone and alum–naltrexone as a novel adjuvant elicits immune responses for Toxoplasma gondii lysate antigen in BALB/c mice. Experimental Parasitology.

[36] Sacerdote P, Gaspani L, Panerai AE (2000). The opioid antagonist naloxone induces a shift from type 2 to type 1 cytokine pattern in normal and skin‐grafted mice. Ann N Y Acad Sci.

[37] Hassan ATM, Hassan ZM, Moazzeni SM, Mostafaie A, Shahabi S, Ebtekar M (2009). Naloxone can improve the anti-tumor immunity by reducing the CD4+ CD25+ Foxp3+ regulatory T cells in BALB/c mice. Int Immunopharmacol.

[38] Rostami H, Ebtekar M, Ardestani MS, Yazdi MH, Mahdavi M (2017). Co-utilization of a TLR5 agonist and nano-formulation of HIV-1 vaccine candidate leads to increased vaccine immunogenicity and decreased immunogenic dose: A preliminary study. Immunol Lett.

[39] Jazani NH, Sohrabpour M, Mazloomi E, Shahabi SJFI, Microbiology M (2011). A novel adjuvant, a mixture of alum and the general opioid antagonist naloxone, elicits both humoral and cellular immune responses for heat-killed Salmonella typhimurium vaccine. FEMS Immunol Med Microbiol.

[40] Caielli S, Veiga DT, Balasubramanian P, Athale S, Domic B, Murat E (2019). A CD4+ T cell population expanded in lupus blood provides B cell help through interleukin-10 and succinate. Nat Med.

[41] Kim ST, Choi J-Y, Lainez B, Schulz VP, Karas DE, Baum ED (2018). Human extrafollicular CD4+ Th cells help memory B cells produce Igs. J Immunol.

[42] Jazani NH, Parsania S, Sohrabpour M, Mazloomi E, Karimzad M, Shahabi SJI (2011). Naloxone and alum synergistically augment adjuvant activities of each other in a mouse vaccine model of Salmonella typhimurium infection. Immunobiology.

[43] Khoury DS, Cromer D, Reynaldi A, Schlub TE, Wheatley AK, Juno JA (2021). Neutralizing antibody levels are highly predictive of immune protection from symptomatic SARS-CoV-2 infection. Nat Med.

[44] Starr TN, Czudnochowski N, Liu Z, Zatta F, Park YJ, Addetia A (2021). SARS-CoV-2 RBD antibodies that maximize breadth and resistance to escape. Nature.

[45] Sauer K, Harris T (2020). An Effective COVID-19 Vaccine Needs to Engage T Cells. Front Immunol.

[46] Cañete PF, Vinuesa CG (2020). COVID-19 makes B cells forget, but T cells remember. Cell.

